# Development of an indirect ELISA for the identification of African swine fever virus wild-type strains and CD2v-deleted strains

**DOI:** 10.3389/fvets.2022.1006895

**Published:** 2022-09-08

**Authors:** Wenting Jiang, Dawei Jiang, Lu Li, Bo Wan, Jiabin Wang, Panpan Wang, Xuejian Shi, Qi Zhao, Jinxing Song, Zixiang Zhu, Pengchao Ji, Gaiping Zhang

**Affiliations:** ^1^College of Veterinary Medicine, Henan Agricultural University, Zhengzhou, China; ^2^International Joint Research Center of National Animal Immunology, College of Veterinary Medicine, Henan Agricultural University, Zhengzhou, China; ^3^Longhu Laboratory, Zhengzhou, China; ^4^Henan Engineering Laboratory of Animal Biological Products, College of Veterinary Medicine, Henan Agricultural University, Zhengzhou, China; ^5^State Key Laboratory of Veterinary Etiological Biology, Lanzhou Veterinary Research Institute, College of Veterinary Medicine, Lanzhou University, Chinese Academy of Agricultural Sciences, Lanzhou, China; ^6^African Swine Fever Regional Laboratory of China, Lanzhou Veterinary Research Institute, Chinese Academy of Agricultural Sciences, Lanzhou, China

**Keywords:** ASFV, CD2v extracellular fragment, CHO cell line, indirect ELISA, identification, CD2v-deleted

## Abstract

African swine fever (ASF) is a potent infectious disease with detrimental effects on the global swine industry and no currently vaccine available. The emergence of low-virulence CD2v-deleted mutants manifested as non-hemadsorption (non-HAD) strains represents a significant challenge to the prevention and control of ASF. In this study, we aimed to establish an indirect ELISA (IELISA) method for the identification of ASFV wild-type and CD2v-deleted strains. We integrated the CD2v protein extracellular domain sequence (CD2v-Ex, 1–588 bp) of the highly pathogenic strain China/2018/AnhuiXCGQ into the genome of suspension culture-adapted Chinese hamster Ovary-S (CHO-S) cells using lentivirus vectors (LVs). By screening, we identified a monoclonal CHO-S cell line that stably expressed secretory CD2v-Ex Protein. We then used the purified CD2v-Ex Protein as the detection antigen to establish an indirect ELISA method (CD2v-IELISA) for identification of the ASFV wild-type and CD2v-Deleted (CD2v^−^) strains. The CD2v-IELISA method showed excellent specificity with no cross-reaction with serum samples infected with ASFV (CD2v^−^), porcine reproductive and respiratory syndrome virus (PRRSV), classical swine fever virus (CSFV), porcine circovirus (PCV), porcine pseudorabies virus (PRV), swine foot and mouth disease virus (FMDV) and porcine epidemic diarrhea virus (PEDV). Furthermore, this method showed high sensitivity, allowing identification of ASFV-infected clinical serum samples up to a dilution of 1:2,560. The coefficient of variation both in and between batches was <10% with good reproducibility and a high compliance rate of 99.4%. This CD2v-IELISA method developed here is of great significance for the prevention, control and purification of ASFV.

## Introduction

African swine fever (ASF) is an acute and severe infectious disease caused by African swine fever virus (ASFV), which can infect domestic pigs and wild boars of all ages and cause up to 100% mortality (1). ASFV is the only currently known insect-borne DNA virus with a genome size of 170–193 kb, encoding more than 150 kinds of proteins although the functions of more than half of these proteins remain to be clarified (2–4). There are currently 24 known genotypes of ASFV based on genetic differences in the protein 72 (p72) capsid protein, and eight serotypes based on hemadsorption inhibition assays (HAI) using ASFV reference immune antisera (5). No effective commercial vaccine is currently available; therefore, effective and accurate methods for laboratory diagnosis are of great significance for the prevention and control of ASFV infection (6, 7).

ASFV was first reported in China in 2018 in an ASF outbreak causing huge economic losses. This strain was identified as p72 genotype II and CD2v serogroup 8, homologous to the highly pathogenic Georgia/2007 strain (8). However, recent studies have shown that ASFV strains with different deletions or mutations of the *EP402R* gene are now spreading. These variant strains show non-hemadsorption (non-HAD) and certain differences in virulence and pathogenicity (reduced pathogenicity but still obvious residues, longer incubation period which can cause persistent infection and chronic disease course, high transmission ability, and marked variation in the clinical manifestations and morbidity between individuals) compared with the earliest isolated highly pathogenic HLJ/2018 strain (9). Consequently, the early diagnosis and monitoring of ASFV has become more difficult. A variety of methods for ASFV antibody detection have been published. Almost all of these methods were used to detect ASFV infection only, but were not able to distinguish infection by ASFV wild-type and CD2v-deleted strains.

ASFV has a complex icosahedral structure (~260–300 nm in diameter) composed of a central nucleoid, core shell, inner membrane, capsid and outer membrane (10). The CD2v protein, encoded by the *EP402R* gene, is a characteristic glycoprotein located in the outer capsule membrane of ASFV. It consists of intracellular, transmembrane and extracellular regions, and it is actively expressed in the late stage of the viral infection process (11, 12). The structure of the CD2v extracellular region is homologous to that of the CD2 protein on the surface of host T cells and natural killer cells. CD2v expression can cause lymphocyte damage, which contributes to the immune escape of the virus. In addition, CD2v binds specifically to CD2 receptors on the surface of porcine red blood cells, leading to a blood adsorption phenomenon and participating in the transport of the virus in the infectious animal (13, 14).

The CD2v protein is an important structural surface antigen of wild-type ASFV. Infection with wild-type ASFV induces the production of specific antibodies that recognize the CD2v protein. Therefore, we used CHO cells to express CD2v as an extracellular protein, which we then used as a detection antigen to establish an iELISA for rapid detection of CD2v antibodies. This assay can be used to distinguish infection by ASFV wild-type strains (HAD) and CD2v-deleted strains (non-HAD) and might be of great importance in epidemiological investigation and normalized monitoring of ASFV.

## Materials and methods

### Plasmid construction and LV production

The CD2v protein extracellular region (GenBank: MK128995.1, CD2v-ex, 1–588 bp) gene sequence was optimized according to the CHO expression system and a 6 × His-tag was introduced at the carboxyl terminus. The recombinant CD2v-ex gene sequence was then cloned into the pTRIP-Pure vector, pTRIP-Pure-CD2v-ex (GenScript, Nanjing, China). Adherent human embryonic kidney (HEK) 293T (ATCC, Manassas, VA, USA) cells were maintained in high glucose Dulbecco's modified Eagle's medium (DMEM; Solarbio, Beijing, China). At 40–50% confluence, HEK 293T cells were seeded in 6-well plates and co-transfected with pTRIP-Pure-CD2v-ex (0.65 μg), psPAX (0.9 μg) and pMDG.2 (0.5 μg) using Lipofectamine 2000 (Invitrogen, Carlsbad, CA, USA); a blank pTRIP-puro vector was used as a control 6–8 h after transfection, the medium was replaced with maintenance medium (DMEM containing 2% FBS). Supernatant containing the pseudotyped LVs was collected at 72 h post-transfection, centrifuged at 4,000 rpm for 10 min at 4°C and filtered through a 0.22 μm filter. The supernatant was then used fresh in experiments or stored at −80°C as previously described (15).

### Establishment of a stable CD2v-ex protein expressing cell clone

CHO-S cells (Thermo Fisher Scientific, Waltham, MA, USA) were passaged in 125-mL vent-cap cell shaker flasks (NEST, Wuxi, China) containing 25 mL ExpiCHO Expression Medium (Gibco, Grand Island, NY, USA) on an orbital shaker platform (100 rpm). When the cell viability exceeded 95%, 2 × 10^6^ cells were mixed with the LV suspension at a ratio of 1:1 (16) in the presence of 5 μg/mL polybrene (Beyotime, Shanghai, China). After 24 h of culture in an orbital shaker at 37°C, the cells were collected by centrifugation at 500 rpm for 10 min at 25°C and resuspended in fresh ExpiCHO culture medium containing puromycin (Beyotime).

The process for screening of a stable cell clone was proceed as follows. Briefly, the transfected CHO-S cells were passaged at a density of 0.5 × 10^6^ viable cells/mL in ExpiCHO culture medium containing 7.5 μg/mL puromycin. When the cell viability exceeded 90%, a second round of screening was performed in ExpiCHO Stable Production Medium (Gibco) containing 22.5 μg/mL puromycin. After 12 h, expression of CD2v-ex by cell clones was evaluated by western blot analysis. Briefly, cell culture supernatants and cell fragmentation supernatants were denatured at 98°C for 10 minand proteins were separated by 7.5% SDS-PAGE before transfer to a polyvinylidene fluoride (PVDF) membrane (Merck Millipore, Billerica, MA, Germany). After blocking for 1 h with 5% skimmed milk (m/v, SM) in tris buffered saline containing 0.05% Tween-20 (TBST, v/v), the membrane was incubated at room temperature (RT) for 1 h with anti-His-tag antibody (1:5,000 in 5% SM, Proteintech, catlog number 66005-1-Ig, Wuhan, China) or standard ASFV-positive serum (1:2,000 in 5% SM, CVCC, Beijing, China). After washing with TBST, the membrane was incubated at 37°C for 1 h with the corresponding secondary antibodies [HRP-conjugated goat anti-mouse IgG (1:5,000 in 5% SM, Proteintech, catlog number SA00001-2) or mouse anti-pig IgG (1:5000 in 5% SM, Immunoway, catlog number RS030232, Plano, TX, USA)]. The antibody-reactive bands were detected using beyoECL star solution (Beyotime) and visualized with a multifunctional imaging system (GE Amersham Imager 600, Boston, Massachusetts, USA).

After two rounds of drug screening, stably transduced cell lines were generated by limiting dilution cloning. The cells were seeded in 96-well plates (0.5 cell per well) with ExpiCHO Expression Medium containing 6 mM L-glutamine (Gibco) and statically incubated at 37°C. Clones expressing high levels of the transfected protein were screened in 24-well and 6-well plates. Finally, the clones expressing the highest levels of the transfected protein was selected in fed-batch culture in 125-mL flasks by feeding with 5, 10, 15 g/L glucose (Gibco) and 2% (v/v) GluMAX-1 (100 ×, Gibco) on days 3, 5, 7 at 37°C. Dot blot and western blot analysis were used to selecting the high-expressing clones (17).

### Genomic verification and protein purification

CHO-CD2v cell lines were verified by measurement of genomic DNA and RNA expression. For DNA verification, genomic DNA was extracted from 1 × 10^7^ stably transduced monoclonal cells using the DNAiso Reagent (Takara, Tokyo, Japan) and identified by PCR amplification. The identification primers were designed based on the optimized gene sequence (CD2v-ex-F: 5'-ATGATCATCCTGATCTTCCTGATC-3', CD2v-ex-R: 5'-TCAGCTGGACAGTGTCAGGTA-3', Tsingke, Beijing, China). The PCR mixture (final volume 20 μL) contained 10 μL 2 × PrimeSTAR MAX Premix (Takara), 1 μL genomic DNA, 1 μL of each primer (10 μM), and 7 μL ddH_2_O. The PCR conditions were set as follows: 98°C pre-denaturation for 30 s; 30 cycles of denaturation at 98°C for 10 s, annealing of 58.5°C for 30 s and extension at 72°C for 30 s; final extension at 72°C for 8 min. The PCR products were identified by 1% (m/v) agarose and sequenced by Beijing Tsingke (Tsingke). For RNA identification, total RNA was isolated using RNAiso Plus (Takara) and cDNA was obtained by reverse transcription using HiScript II One Step qRT-PCR Probe Kit (Vazyme, Shanghai, China). The following PCR operations were the same as the above mentioned.

To prepare purified CD2v-ex protein for subsequent tests, the selected CHO-CD2v cell line was cultured for 7–10 days in a shaking incubator (100 rpm) and fed with 5 g/L glucose (Gibco) and 2% (v/v) GlutaMax-1 (100 ×, Gibco) every 2 days. After centrifugation with 12,000 × *g* for 10 min, the supernatant was collected and CD2v-ex protein was purified from the cell culture supernatant using HisTrap excel (Cytiva, Sweden). The purified protein was analyzed by SDS-PAGE.

### Indirect ELISA method development

CD2v-ex protein was diluted with carbonate bicarbonate buffer (CBS buffer, pH 9.6) and used to coat 96-well plates (Beaver, Suzhou, China) at 4°C overnight. The plates were then washed five times with TBST and patted dry. After the plates were blocked for 1 h at RT with 5% SM (in TBST) and washed as above, serum samples were diluted with 1% bovine serum albumin (1% BSA, m/v) in 1 × phosphate buffered saline (PBS) and then added to plates (100 μL/well) and incubated for 30 min at 37°C. Subsequently, the plates were washed as above and incubated with HRP-conjected monoclonal mouse anti-pig IgG (Immunoway) diluted to 1:10,000 with 5% SM. After incubation for 40 min at 37°C and washing, the plates were incubated with 3,3′,5,5′-tetramethylbenzidine (TMB, Solarbio) at RT. The reactions were stopped after 10 min by adding 2 mol/L H_2_SO_4_ (50 μL/well). The optical density (OD) of each well was measured at 450 nm (OD_450_) using a Multimode Microplate Reader (Tecan 10M, Switzerland).

The antigen and serum concentrations were optimized by checkerboard titration. Briefly, 96-well plates were coated with the recombinant antigen titrated to concentrations of 0.5–4 μg/mL (50–400 ng/well). ASFV-positive (ASFV^+^) and ASFV-negative (ASFV^−^) standard sera were serially diluted 1:10–1:320. Every combination was evaluated in duplicate.

The CD2v-iELISA reaction conditions were optimized according to the optimal antigen coating concentration and serum dilution determined as previously described. The optimal assay conditions were identified as those that yielded the highest OD_450_ ratio between the ASFV^+^ and ASFV^−^ serum samples (P/N value) as previously described (18). Every sample was evaluated in triplicate.

The status of 82 swine ASFV^−^ serum samples stored in our laboratory was first confirmed using the P30-iELISA kit (Kernal, US) according to the manufacturer's instructions. These 82 serum samples were then analyzed using the optimized CD2v-iELISA method. The cut-off value was defined as the mean OD_450_ value + 3 × the standard deviation (SD), and samples above this cut-off value were considered to be ASFV^+^ (19).

### Determination of the specificity, sensitivity, reproducibility, and compliance rate

The specificity of the established CD2v-iELISA method was determined against the following serum samples: ASFV^+^ (positive control), ASFV^+^ with CD2v deleted (CD2v^−^), porcine reproductive and respiratory syndrome virus positive (PRRSV^+^), classical swine fever virus positive (CSFV^+^), porcine circovirus positive (PCV^+^), porcine pseudorabies virus positive (PRV^+^), swine foot and mouth disease virus positive (FMDV^+^) and porcine epidemic diarrhea virus positive (PEDV^+^). The OD_450_ of each pathogenic serum sample was compared with the cut-off value and samples above this cut-off value indicated a cross-reaction.

The sensitivity of the CD2v-iELISA was evaluated using three clinical ASFV^+^ and three ASFV^−^ serum samples diluted from 1:160 to 1:5,120. The assay sensitivity correlated with the highest dilution of serum detectable according to the cut-off value.

Eight ASFV^+^ positive and/or ASFV^−^ serum samples were randomly selected. Triplicate samples were assayed in one batch to evaluate intra-assay variation and in three different batches were assayed separately to evaluate inter-assay variation expressed as the coefficient of variation (CV).

A total of 179 clinical swine ASFV^+^ (*n* = 18), ASFV^−^ (*n* = 112) and ASFV^+^ (CD2v^−^) (*n* = 49) serum samples determined by real-time quantitative PCR (qPCR) and HAD tests were kindly provided by Lanzhou Veterinary Research Institute and analyzed using the established CD2v-ex-iELISA method to determine the compliance rate.

### Statistical analysis

All statistical analysis was conducted using GraphPad Prism 8.2 software (Graph Pad Prism Inc., California, USA). Data were presented as the mean + SD. Significant differences between samples were assessed using Student's *t*-test. *P* < 0.05 was set as the threshold for statistical significance.

## Results

### Establishment of a stable CD2v-ex protein expressing cell clone

**Figure 1:**
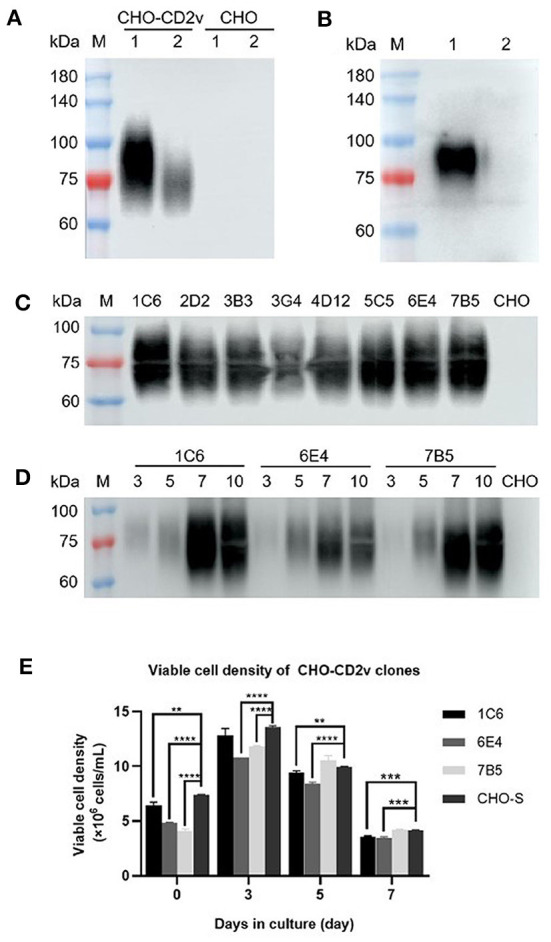
Establishment of a stable CD2v-ex protein expressing cell clone. Western blot detection of CD2v-ex protein expression after transduction of CHO-S cells using an anti-His-tag antibody: Lane 1: cell culture supernatant, Lane 2: cell fragmentation supernatant **(A)**. Western blot detection of CD2v-ex protein expression using standard ASFV^+^ serum **(B)**. Western blot comparison of expression levels of CHO-CD2v monoclonal cell lines (1C6, 2D2, 3B3, 3G4, 4D12, 5C5, 6E4, 7B5) after culture for 5 days in a 6-well plate **(C)**. Western blot detection of CD2v-ex protein in culture supernatants of three high-expression cell clones (1C6, 6E4, 7B5) collected on days 3, 5, 7, 10 **(D)**. Viable cell density of three high-expression CHO-CD2v clones after 3, 5, 7, 10 days in culture. **p < 0.01, ***p < 0.0001, and ****p < 0.00001 **(E)**. Blank CHO-S cells were treated as a negative control; M: Protein marker 2,510.

To establish a stable CD2v-ex protein expressing CHO-S cell line, CHO-S cells were first transduced with packaged LV suspensions to generate CHO-S cells expressing CD2v-ex (CD2v-ex) and CD2v-ex expression was verified by western blot analysis. A specific diffuse band (70–95 kD) was detected in CHO-CD2v cell culture supernatant (Figures 1A,B), consistent with eukaryotic expressed glycosylated proteins' characteristics (16, 20), suggesting that CHO-CD2v cells successfully secreted express glycosylated CD2v-ex. Furthermore, the protein was recognized by anti-His-tag antibody and ASFV^+^ serum. To select a stable CD2v-expressing CHO cell line, monoclonal cells were obtained by limiting dilution cloning and then successively selected and expanded from 96-well plates (*n* = 92), to 24-well plates (*n* = 26) and finally, to 6-well plates (*n* = 8, 1C6, 2D2, 3B3, 3G4, 4D12, 5C5, 6E4, 7B5). Three high-expression CHO-CD2v cell clones (1C6, 6E4, 7B5) were then selected (Figure 1C), and the highest expression monoclonal cell line in fed-batch culture was identified as 1C6 by western blot analysis (Figure 1D). In addition, viable cells monitoring revealed that 1C6 showed a comparable growth curve compared with blank CHO-S cells in fed-batch culture (Figure 1E), suggesting that 1C6 was a high-expressing CHO-CD2v clone with growth characteristics similar to those of the parent line.

### Genomic verification and protein purification

**Figure 2:**
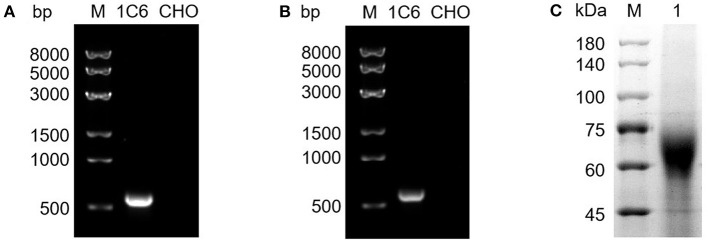
Genomic verification and protein purification of a high-expression CHO-CD2v cell line. The total genomic DNA and RNA of 1C6 cell line were extracted from the 1C6 cell line and the presence of the CD2v-ex at the genomic DNA and mRNA levels confirmed by PCR **(A)** and RT-PCR **(B)** analyses, respectively; blank CHO-S cells were treated as a negative control. SDS-PAGE analysis of purified CD2v-ex protein **(C)**.

For further verification of the selected CHO-CD2v clone (1C6), we extracted its genomic DNA and RNA, respectively. Integration of the CD2v-ex gene sequence into CHO-S cell genomic DNA was confirmed by PCR and transcription into mRNA for protein expression was verified by RT-PCR, as evidenced by detection of a specific band of the expected size (588 bp) (Figures 2A,B), and the sequencing results were correct. The CD2v-ex protein was purified from cell culture supernatant using HisTrap excel (Cytiva) in binding buffer (20 mM Tris-HCl,300 mM NaCl, pH 8.0) to purified it from cell culture supernatant. CD2v-ex was eluted from the column with the same binding buffer supplemented with 75 mM imidazole and SDS-PAGE confirmed the presence of the purified CD2v-ex protein (Figure 2C).

### Determination of the CD2v-IELISA reaction conditions

**Figure 3:**
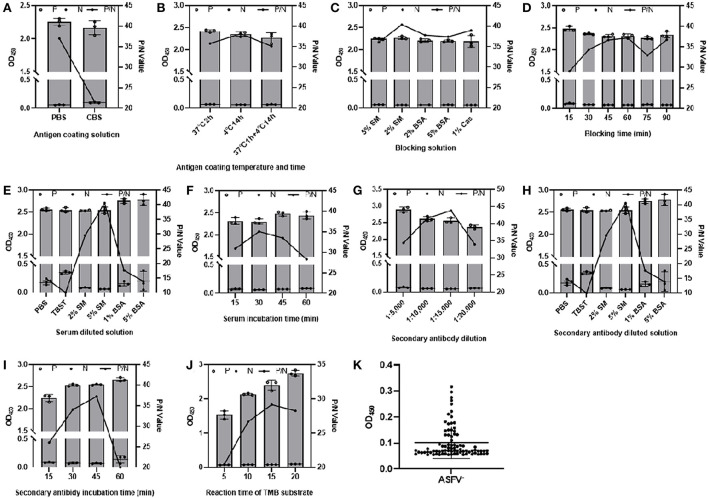
Optimization of CD2v-iELISA conditions. The CD2v-iELISA method was optimized by changing the following parameters: antigen coating solutions **(A)**, antigen coating temperature and time **(B)**, blocking solution **(C)**, blocking time **(D)**, serum dilution **(E)**, incubation time **(F)**; secondary antibody dilutions **(G)**, secondary antibody dilution factor **(H)**, secondary antibody incubation time **(I)**, TMB substrate incubation time **(J)**. P and N value data represent mean ± SD and P/N value data represent the ratios of positive mean value (P) to negative mean value (N). A total of 82 negative sera were analyzed to determine the cut-off value **(K)**.

To determine the optimal reaction conditions of CD2v-iELISA, we first identified the optimal coating antigen concentration and serum dilution factor by checkerboard titration, with the coating antigen concentration at 0.5–4 μg/mL and serum dilution at 1:10–1:320. The maximum positive/negative (P/N) value was obtained when the concentration of CD2v-ex protein was 2 μg/mL (200 ng/well) and the serum dilution was 1:160 (Table 1). Using the same criterion of the conditions that yield the maximum P/N value, the remaining reaction conditions were successfully optimized (Figures 3A–J) as described in Table 2. The cut-off value was determined using the optimal CD2v-iELISA method to analyze 82 ASFV^−^ serum samples. The mean OD_450_ value was 0.1017 and the SD was 0.0616 (Figure 3K), resulting in a cut-off value of 0.2865 (0.287, mean + 3 × SD). Therefore, only samples with OD_450_ values ≥0.287 were considered to be ASFV^+^; all others were classified as ASFV^−^. negative.

**Table 1:** Determination of the optimal reaction conditions of the CD2v-iELISA.

**Antigen concentration (**μ**g/mL)**		**Serum dilution**					
		**1:10**	**1:20**	**1:40**	**1:80**	**1:160**	**1:320**
4	P^a^	2.740	2.673	2.635	2.580	2.549	2.455
	N^a^	0.942	0.588	0.328	0.209	0.177	0.192
	P/N^b^	2.910	4.547	8.041	12.347	14.429	12.805
2	P	2.736	2.561	2.584	2.553	2.524	2.335
	N	1.119	0.666	0.346	0.167	0.113	0.119
	P/N	2.445	3.844	7.465	15.266	22.429	19.665
1	P	2.646	2.599	2.606	2.507	2.456	2.256
	N	1.220	0.729	0.307	0.213	0.140	0.145
	P/N	2.169	3.564	8.491	11.777	17.485	15.611
0.5	P	2.337	2.318	2.392	2.300	1.403	1.102
	N	1.282	0.775	0.401	0.222	0.144	0.137
	P/N	1.823	2.993	5.966	10.354	9.729	8.046

a Each data point represents the mean of two replicates.

b P/N value data represent the ratios of positive mean value (P) to negative mean value (*N*).

**Table 2:** Optimized conditions of the CD2v-iELISA.

**Reaction conditions**	**Evaluation options**	**Optimized results**
Antigen coating solution	PBS (pH 7.2), CBS (pH 9.6)	PBS (pH 7.2)
Coating temperature and time	37°C 2 h, 4°C overnight, 37°C 1 h and then 4°C overnight	4°C overnight
Blocking solutions	2% SM, 5% SM, 2% BSA, 5% BSA, 1% casein (Cas)	2% SM
Blocking time	30, 45, 60, 75 and 90 min	60 min
Serum diluent	PBS, TBST, 2% SM, 5% SM, 1% BSA and 5% BSA	1% BSA
Serum incubation time	15, 30, 45 and 60 min	30 min
Secondary antibody dilution	PBS, TBST, 2% SM, 5% SM, 1% BSA and 5% BSA	5% SM
Secondary antibody dilution factor	1:5000, 1:10,000, 1:15,000 and 1:20,000	1:15,000
Secondary antibody incubation time	15, 30, 45 and 60 min	45 min
TMB substrate reaction time	5, 10, 15 and 20 min	15 min

SM was diluted in TBST (m/v), BSA and Cas was diluted in PBS (m/v), FBS was diluted in PBS (v/v).

### Determination of the CD2v-IELISA specificity, sensitivity, reproducibility, and compliance rate

**Figure 4:**
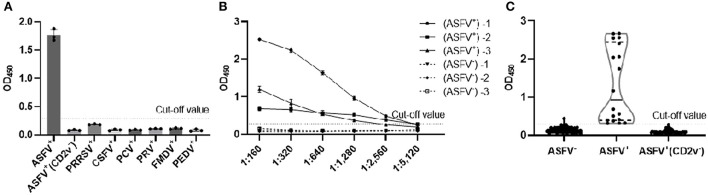
Determination of the specificity, sensitivity, and compliance rate of the CD2v-iELISA. The CD2v-iELISA did not detect ASFV^+^(CD2V^−^), PRRSV^+^, CSFV^+^, PCV^+^, PRV^+^, FMDV^+^ and PEDV^+^ serum samples at 1:160 dilution **(A)**. Three clinical ASFV^+^ and three ASFV^−^ serum samples were diluted from 1:160 to 1:5,120 to determine the highest dilution of serum detected in the assay according to the cut-off value **(B)**. The CD2v-iELISA was used to detect ASFV^+^ (*n* = 18), ASFV^−^ (*n* = 112), ASFV^+^ (CD2v^−^) (*n* = 49) serum samples to determine the assay compliance rate of the assay according to the cut-off value **(C)**.

To determine the CD2v-iELISA method specificity, we analyzed ASFV^+^(CD2v^−^), PRRSV^+^, CSFV^+^, PCV^+^, PRV^+^, FMDV^+^ and PEDV^+^ serum samples; ASFV^+^ was used as the positive control. All pathogen positive sera tested negative with the exception of the ASFV^+^ control (Figure 4A), indicating excellent assay specificity.

To determine the CD2v-iELISA sensitivity, we analyzed three clinical ASFV^+^ and three ASFV^−^ serum samples (diluted from 1:160 to 1:5,120) using the determined cut-off value. The highest dilution of the ASFV^+^ sample that was detected in the assay was 1:2,560 (Figure 4B), indicating an assay sensitivity up to 1:2,560.

The reproducibility of the CD2v-iELISA was determined based on intra- and inter-assay CV values calculated by analyzing eight randomly selected ASFV^+^ and/or ASFV^−^ serum samples in a single batch and three different batches, respectively. As shown in Table 3, the intra- and inter-assay values were both <10%, suggesting a high repeatability and low variability.

**Table 3:** CD2v-ex-iELISA repeatability.

**Sample number**	**Intra-assay repeatability**		**Inter-assay repeatability**	
	**Mean ±SD^a^**	**CV^b^**	**Mean ±SD^a^**	**CV^b^**
1	0.686 ± 0.040	5.80	1.372 ± 0.023	1.67
2	0.876 ± 0.035	3.94	1.148 ± 0.041	3.56
3	1.865 ± 0.039	2.07	2.325 ± 0.068	2.91
4	0.089 ± 0.006	6.37	0.076 ± 0.004	5.20
5	0.087 ± 0.005	5.21	0.111 ± 0.009	8.38
6	0.183 ± 0.011	6.11	0.250 ± 0.021	8.29
7	0.076 ± 0.007	9.62	0.103 ± 0.009	8.89
8	0.103 ± 0.008	8.14	0.115 ± 0.010	9.00

a Each data point represents the mean of three replicates.

b CV (coefficient of variation) = SD/Mean × 100%.

To determine the CD2v-iELISA compliance, was analyzed ASFV^+^ (*n* = 18), ASFV^−^ (*n* = 112), and ASFV^+^ (CD2v^−^) (*n* = 49) serum samples. The assay showed 100% sensitivity and specificity in identification of ASFV^+^ and ASFV^+^ (CD2v^−^) serum samples, and the total compliance rate was 99.4% (Figure 4C).

## Discussion

ASFV was first reported in Kenya in 1921 (21) and reached Europe in the 1950s, where it persisted and became endemic (22–24). China was the first country in Asia to report the occurrence of ASF, which subsequently spread to 16 Asian countries including Korea, Japan, and Vietnam by 2021. Since 2005, ASF has been reported in 73 countries around the world according to the World Organization for Animal Health (OIE), resulting in huge economic losses to the pig industry. Laboratory assays are designed primarily to detect nucleic acids, antigens and antibodies. The ASFV detection tests recommended by the OIE include virus isolation, fluorescent antibody testing (FAT), and real-time and routine PCR testing, which is the most widely used technique at the EU National Reference Laboratory (NRL) level (25). Although the ELISA method is not as sensitive as PCR, it has important application value due to its ease of operation and potential for analysis of large-scale samples (26).

The CHO cell expression system is widely used in recombinant protein expression because of its ability to maintain appropriate post-translational modifications, and glycosylation in particular (27). Furthermore, recombinant protein expression can be improved by integrating the target gene into the host genome to generate a stable cell line (28). CHO cells are used most widely used in mammalian expression systems involved in the production of >70% of recombinant biopharmaceutical proteins (29). After genetic modification and domestication, several modified CHO cell lines, such as CHO-K1, CHO-S, and CHO-DG44, have been modified to generate genetically stable systems for the production of a variety of different types of proteins (30). The CHO-S cell line was adapted to suspension culture and use in large-scale production of recombinant proteins.

Blocking ELISA (bELISA) and iELISA methods are used for the detection of ASFV-specific antibodies generated against important structural antigens such as protein 30 (p30), protein 54 (p54) and p72 (31, 32). Among the commercial ASFV ELISA kits currently available, the Ingenasa-bELISA is based on p72 (Ingenasa, Spain), while the IDvet-iELISA is based on protein 32 (p32), protein 62 (p62) and p72 (ID-vet, France), and the Svanovir-iELISA is based on p30 (Svanovir, Sweden).

With the prevalence of low-force ASFV CD2v mutants, a detection method that can be used to distinguish wild-type ASFV from CD2v-deleted strains of ASFV is urgently required for effective monitoring. In previous research, a specific quantum dot-based immunochromatographic assay was developed using CD2v as the diagnostic antigen to detect ASFV antibodies with a titer of 1:5.12 × 10^5^ and a compliance rate of 85.92% (33). A triplex real-time PCR assay that was also successfully established to identify pigs infected with wild-type ASFV strains and an ASFV double-gene deletion strain (MGF360-505R and/or CD2v), was completely consistent with the OIE-recommended assay (34). In this study, the CD2v protein was truncated and the extracellular domain was selected for expression with a signal peptide without an intracellular cell-penetrating peptide (CPP), which not only retained the key domain involved in blood adsorption, but also improved protein secretion and expression (35). The CHO-CD2v stable cell line established in this study not only expressed a protein that was similar to the natural conformation, but also ensured stable and high-level expression of the protein of interest. Compared with transient transfection, this approach is significantly more cost-effective. The recombinant CD2v-ex protein was used as a coating antigen to establish and optimize the ASFV CD2v specific antibodies detection method, showing a pretty high concordance rate of 99.4 (178/179), and it was more applicable to the detection of large-scale samples than the PCR method.

In this study, we established an iELISA method using CD2v-ex protein as a coating protein for rapid, specific and sensitive diagnosis of ASFV CD2v antibodies. Compared with commercial ELISA kits coated with conserved ASFV antigens (such as p30, p54, p72), this CD2v-iELISA provides a method for specific identification of ASFV wild-type and CD2v-deleted strain infections. Therefore, we propose that the application this CD2v-iELISA with the traditional ASFV detection method will have more effective diagnostic value because of its negative results of ASFV^+^(CD2v^−^) serum samples.

## Data availability statement

The original contributions presented in the study are included in the article/supplementary material, further inquiries can be directed to the corresponding author/s.

## Author contributions

WJ and DJ contributed to design and perform the majority of experiments. WJ draft the manuscript. LL and BW carried out the western blot and dot blot analysis. JW, PW, and XS was responsible for PCR and RT-PCR identifications. QZ, JS, and ZZ analyzed data. PJ contributed to revising the work critically for important intellectual content. PJ and ZZ provided approval for publication of the content and agreed to be accountable for all aspects of the work in ensuring that questions related to the accuracy or integrity of any part of the work are appropriately investigated and resolved. All authors read, contributed to, and approved the final manuscript.

## Funding

This study was funded by the National Natural Science Foundation of China (grant number: 31941001) and the Key Scientific Research Project of Colleges and Universities of Henan Province (grant numbers: 22B230006 and 21A230010).

## Conflict of interest

The authors declare that the research was conducted in the absence of any commercial or financial relationships that could be construed as a potential conflict of interest.

## Publisher's note

All claims expressed in this article are solely those of the authors and do not necessarily represent those of their affiliated organizations, or those of the publisher, the editors and the reviewers. Any product that may be evaluated in this article, or claim that may be made by its manufacturer, is not guaranteed or endorsed by the publisher.
